# Cross-Layer Routing for a Mobility Support Protocol Based on Handover Mechanism in Cluster-Based Wireless Sensor Networks with Mobile Sink

**DOI:** 10.3390/s19132843

**Published:** 2019-06-26

**Authors:** Maamar Zahra, Yulin Wang, Wenjia Ding

**Affiliations:** 1 School of Computer Science, Wuhan University, Wuhan 430072, China; 2 Shenzhen Research Institute, Wuhan University, Shenzhen 518057, China

**Keywords:** cross-layer, MAC layer, network layer, mobility model, cluster, mobile sink, handover, prediction, mobility management

## Abstract

Wireless sensor networks with mobile collectors or sinks face some challenges regarding the data collection process and the continuous connectivity and delivering of data while the mobile sink is moving throughout the network. These challenges increase as the network grows. For this aim, we propose in this paper a cross-layer routing protocol which supports mobility for large-scale wireless sensor networks, which we name CLR-MSPH. We adapt CLR-MSPH for the hierarchical architecture of the network, and it performs on cluster-based wireless sensor networks where the network is organized in clusters. Our proposed protocol deals with the problem of handover data after the mobile sink leaves the radio range of cluster head without sending all data stored in the cluster head’s buffer. We also introduce a mobility model for the mobile sink for a better data collection process. CLR-MSPH is considered as an extending implementation of BMAC protocol with handover mechanism (BMAC-H). In order to prove the efficiency of the proposed protocol, we compare CLR-MSPH to BMAC-H, where we adapted BMAC-H to perform in cluster-based wireless sensor networks. The simulation results show that CLR-MSPH performs better than BMAC-H in terms of packets reception rate, energy, and latency.

## 1. Introduction

The primary task in wireless sensor networks is data collection. Recently, many research works have investigated the data collection process, especially for large-scale networks, to address the challenges related to this process, such as energy consumption, connectivity, and latency. This primary task leads researchers to find practical solutions such as introducing mobility to this kind of networks [1,2,3,4,5,6,7,8,9], because of the great revolution in robotics that may be useful for wireless sensor networks to solve problems related to connectivity, routing and data collection [10]. The mobility in wireless sensor networks also solves the problem of the quick energy dissipation of nodes in the vicinity of the sink in the traditional wireless sensor networks with static sinks due to their role in forwarding data to the sink [11]. However, by making a collector node a mobile node we can help distribute the energy consumption in the network. In this study, we assume that the concerned mobile node is the sink. The mobile sink (MS) moves throughout the network and passes by sensor nodes to collect the sensed data from them. The strategy of using mobility in wireless sensor networks improves the connectivity of the network by reaching the isolated nodes. 

For large-scale wireless sensor networks, many research works propose dividing the network into clusters (cluster-based wireless sensor networks). Each cluster is composed of one cluster-head (CH) and a group of cluster members (CMs) [12,13,14]. CMs send the sensed data to their CH. When the MS arrives at the CH vicinity, the CH transfers or forwards the sensed data of its members to the MS. The aim of using a cluster-based wireless sensor network is to reduce the energy dissipation in the network, and thus to extend the network lifetime. Besides, using the cluster-based architecture helps to reduce the latency of the tour length of the MS in the network by visiting only CHs rather than visiting all sensor nodes. In addition, the trajectory length of the MS is considered a factor which affects the latency of data collection [15].

When the MS enters the radio range of a CH, the CH starts transferring data to the MS. In some cases, due to the speed of the MS taking into consideration the amount of data to be transferred from the CH to the MS, the CH can estimate that the transfer of data will not be completed in one single hop communication with the MS. Therefore, a continuous connectivity mechanism must be integrated into the transmitter node, which is the CH, to route the data to the receiver, which is the MS while it is moving. To do so, a handover mechanism is proposed for cluster-based wireless sensor networks. The handover mechanism is widely used in cellular networks to ensure the connectivity and the continuity of delivering data in a session between the transmitter and receiver without data loss and interruption, in the case where the channels are changing [16].

Introducing the handover mechanism in cluster-based wireless sensor networks to route the data, is considered a challenge because of the hierarchical architecture of the network and the need to control the handover mechanism by enabling some specific nodes to perform the handover process. In addition, some other points must be considered in this kind of networks such as the data delivery rate, energy, the speed of the MS, the end-to-end delay, the handover process delay and its influence on the end-to-end delay.

In this paper, we propose a cross-layer routing for mobility support protocol based on a handover mechanism in multi-hop communication (CLR-MSPH for short). Our proposed protocol is based on predicting the suitable relay nodes depending on the trajectory of the MS. The design of the proposed mobility support protocol CLR-MSPH allows the network layer and the MAC layer to cooperate and exchange information messages to ensure a better routing and handover mechanism in the cluster-based wireless sensor network. Our proposed CLR-MSPH is originally based on the BMAC protocol. In order to prove the efficiency of CLR-MSPH, we compare CLR-MSPH to the original MAC protocol BMAC which we adapt to the cluster-based wireless sensor network scenario to process handover. We name it for short BMAC-H. The main contributions in this paper are as follows:

(1) Design a routing protocol based on handover mechanism for cluster-based wireless sensor network with MS in multi-hop communication (CLR-MSPH),
(2) Introduce a handover control mechanism for CLR-MSPH in order to improve data collection,
(3) Adapt the handover mechanism with BMAC protocol for cluster-based wireless sensor network (BMAC-H),
(4) Adapt a mobility model based on metaheuristic method in order to optimize the movement of the MS in cluster-based wireless sensor networks.

The remainder of this paper is organized as follows: in the next section, we present the study problem of this work. In Section 3, we cite previous works which have dealt with the problem of handover mechanisms in WSNs. We also present the mobility model used for the MS in details in Section 4. In Section 5, we introduce the design of the proposed CLR-MSPH and the adapted BMAC-H in details. Section 6 shows the results of simulating both the CLR-MSPH and BMAC-H protocols in order to compare their performance. Finally, we conclude the paper in Section 7.

## 2. Problem and Motivation

The major challenge in cluster-based wireless sensor networks with MS is finding suitable relay nodes to route data from CH to the MS when the latter leaves the radio range of the CH, especially in such hierarchical architecture of network and characteristics of nodes. The moment when the MS is about to leave the radio range of the CH, the CH activates the handover process to find an attachment or relay node to forward data for the sake of continuous communication and delivering of data. In the literature, research works consider the handover process in the classical wireless sensor network, and there are a few works that take into consideration the handover mechanism in hierarchical wireless sensor networks such as cluster-based wireless sensor networks. The problem in cluster-based wireless sensor networks consists of the nature and characteristics of nodes in the network where only CHs are allowed to send data to the MS. CMs are only allowed to send and route data to their CH, so it is a matter of organizing the data transfer policy in order to avoid the congestion of the network. In order to process the handover mechanism, a CM might be selected as a relay node to route data to the MS, which requires an update of the data transfer policy of this node. 

As we are interested mainly in the problem of transferring data from CHs to the MS directly or using some relay nodes, we do not focus much on sending data from CMs to CHs. In this work, we base on the clustering protocol LEACH [17] for the phase of forming clusters, selecting CHs and route data from CMs to CHs where we are interested in the packets exchanged in the cluster-forming phase, such as the advertisement packet and the joint packet which are sent by the CH and CMs, respectively. The LEACH protocol is based on a random rotation mechanism to select cluster heads in order to distribute the energy consumption of the network. However, for the sake of objectivity of our work and simplicity, we study the impact of handover mechanism on clusters in one rotation round of cluster heads in terms of energy consumption, data collection, and latency which is the scope of our work. We also assume that the MS knows the network topology and CHs positions in order to optimize the trajectory of data collection. 

In addition, we assume that the MS informs CHs by its next destination using the allow sending packet. The allow sending packet is sent by the MS to the CH to make the CH start sending data stored in the buffer. The CH uses the information of the next destination to estimate the relay node in order to active the handover process in the case of the communication between it and the MS ends up without sending all the stored data.

The CH detects that the MS approaches to leave the communication area of CH by measuring the RSSI value of the ACK message. The CH sends data packets to the MS, and the latter sends back ACK message to confirm the reception of data packets. Each ACK message received by the CH, has an RSSI value. CH compares this value to the threshold (we set the RSSI threshold to −90 dBm). If the RSSI of the received ACK is less or equal to the RSSI threshold, CH knows that the MS is leaving, and it starts the handover mechanism.

**Figure 1:**
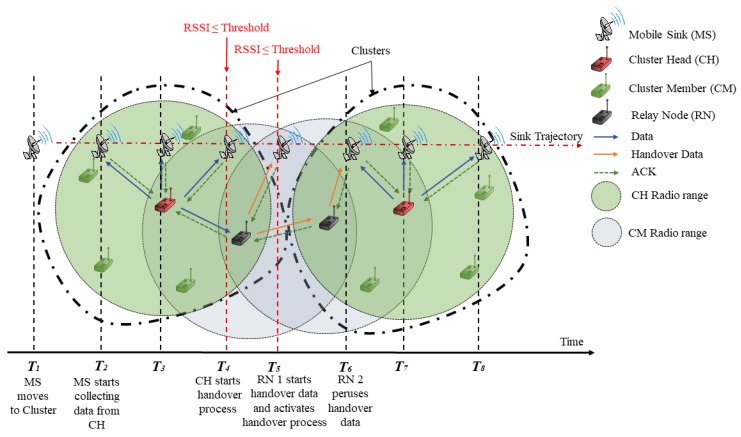
Proposed Handover Mechanism CLR-MSPH in Cluster-Based Wireless Sensor Network.

Figure 1 shows the studied problem of the handover mechanism in cluster-based wireless sensor networks and illustrates the main idea of the proposed protocol CLR-MSPH. When the MS leaves the radio range of CH without receiving all data from CH. CH selects relay nodes (RN) to activate the handover process in order to route data to the MS. 

## 3. Related Work

Recently, mobility in wireless sensor networks has attracted significant interest, especially with the evolution of mobile robotics and smart mobile robots [10]. In addition, mobility is considered one of the essential solutions for energy consumption and improvement of the data collection process in this kind of network [1,15,18].

In the literature, there are three mobility types for nodes in the networks, which are the mobility of sensor nodes, mobility of collectors or sinks, and the mobility of sensor nodes and collectors together. As we mentioned before, we are interested in the mobility of the collector node or the sink, which is named mobile sink (MS) in this paper. 

For large-scale networks, clustering is proposed to organize the wireless sensor network [12,13,14]. In the cluster-based wireless sensor network, CHs receive data sensed by sensor nodes in a single hop then the MS collects these data directly from CHs. To do so, the MS needs a trajectory plan to pass by all CHs in the network and gather data. Few works dealt with the problem of finding the best path to collect data in wireless sensor networks [15,19,20,21].

The moving plan of MS through the network field is considered as a problem in [15,19,21,22], where they refer this problem to the well-known problem traveling salesman problem (TSP) which is considered an NP-hard class problem. These research works studied the problem of finding an optimal path for the MS using optimization methods such as metaheuristics algorithms for the sake of improving the data collection process and extend the network lifetime. In this paper, we use a mobility model based on metaheuristic algorithms used in a previous publication [15], in which MS collects data from each sensor nodes in the network. We adapt this mobility model to cluster-based wireless sensor networks in order to make MS collects data directly from CHs in order to reduce latency. This mobility model gave significant results regarding data collection and energy saving.

While the MS is moving through the network field, interruption of communication may occur between the MS and CHs. To handle this problem, the handover mechanism is proposed. Many works in the literature such in [4,23,24,25,26], study the handover mechanism in the classical wireless sensor networks and a few of them address the handover problem in the hierarchical wireless sensor networks such as cluster-based wireless sensor networks. Most of these research works deal with the handover at the MAC layer where they propose seamless handover mechanism based on some existed MAC protocols such as BMAC [24], XMAC [23], MA-MAC [24], SMAC [27] and RI-MAC [26]. 

In [23], authors proposed a seamless handover based on XMAC protocol at the MAC layer for a classical wireless sensor network with MS, named in this paper HXMAC. The source node starts sending the data stored in the buffer to the receiver (MS). The MS replies by an acknowledgment (ACK) to the source node. Meanwhile, the mobility estimation scheme at the background evaluates the RSSI values of the received ACK packets. The initiation of the handover in the proposed HXMAC depends on the value of the RSSI, the speed of the MS, and the minimum distance between the source node and the receiver [26]. The handover mechanism used in HXMAC starts by broadcasting a handover request and waits for a handover response from other sensor nodes in the network. The source node selects the closest node which replied with a handover response in order to perform the handover and route data packets in two hop routing. The mobility model used in this paper is the random waypoint model with varying the speed of the MS from 1 m/s to 5 m/s.

The minimum distance between the source node and the receiver used by the MS in [23], in order to initiate the handover process, is defined in Equation (1) [26]:

(1) $$d=R-\left(n-w\right)\left(\frac{N_{data}}{R_{t}}+\frac{N_{b}}{R_{t}}+2T_{SIFS}\right)v$$

where *R* is the radio range, n is the number of data packets to be transmitted. Among these data, w is the first acknowledged packets. (n−w) data packets are left for transmission. $N_{data}$, $N_{b}$, $R_{t}$ and $T_{SIFS}$ represent respectively the data packet size, the ACK beacon size, the transmission rate and the time necessary to switch from transmitting to receiving mode. Finally, v represents the velocity of movements.

The authors in [24] propose the mobility aware MAC (MA-MAC) protocol in classical wireless sensor networks. MA-MAC is an extending implementation of any energy efficiency protocols such as BMAC and XMAC. This proposed protocol is implemented in all mobile nodes in the network, and it evaluates the acknowledgments packets in order to determine the quality of transmission using the RSSI values. If the case of a deterioration of RSSI value, the handover initiates by switching the transmission of packets from unicast mode to broadcast mode. A neighboring discovery is launched while broadcasting data packets. Once a neighbor node replies the neighboring discovery, the mobile node establishes a link with this node and switches again to unicast mode then this node starts sending the handover data. The mobility model used to study MA-MAC protocol is based on the human activities and movements with taking into consideration the slow speed of movements of people, which is 1.5 m/s.

In [27], the authors propose a handover mechanism based on SMAC protocol for cluster-based wireless sensor networks. Each cluster in the wireless sensor network shares a schedule for transmitting data which is similar to SMAC protocol. The paper applies a small modification in the scheduling process, which is the Node-Type flag. By the end of this process, each cluster is divided into three types: cluster head, border node, and stationary node. The detection of the deterioration of links in the proposed handover mechanism is based on the RSSI value and link quality. In the case of degradation of link quality, a stationary node changes its flag to a mobile node. While the mobile node is moving, it receives a synchronization packet from the border node. When it receives this packet, it sets a handover-bit in its synchronization packet and broadcasts it to process the handover. The border node also broadcasts the synchronization packet, and it includes the schedule of the neighbor cluster in this packet. Thus, the mobile node can receive a copy of the neighbor cluster schedule and adopts its schedule and the schedule of the neighbor cluster as well. In this case, the mobile node acts like a border node, which allows handover process to another cluster. The mobile nodes use a random walk mobility model varying their speed from 0.5 m/s to 20 m/s.

A zone-based routing mobile sensor network (Zoro MSN) is proposed in [28]. The network is composed of zones, and each zone has a zone head, and any node can be a mobile node. Routing data using the proposed protocol is based on the discovery process of zone heads to find nodes in its vicinity in order to route data collected to other zones till data arrive at the base station (sink) which is considered as static. The mobility model used in this protocol is a random model with a random variation in the speed of the mobile nodes.

There are also some research works that propose mobility support protocols with a handover mechanism based on 6LoWPAN technology [4,29]. The main idea of 6LoWPAN technology is proposing an adaptation layer between the link layer and the network Layer to address the problem of IPv6 packets size compared to IEEE 802.15.4 frame size used in wireless sensor network with taking into consideration the characteristics of wireless sensor network.

## 4. Mobility Model of the MS

**Figure 2:**
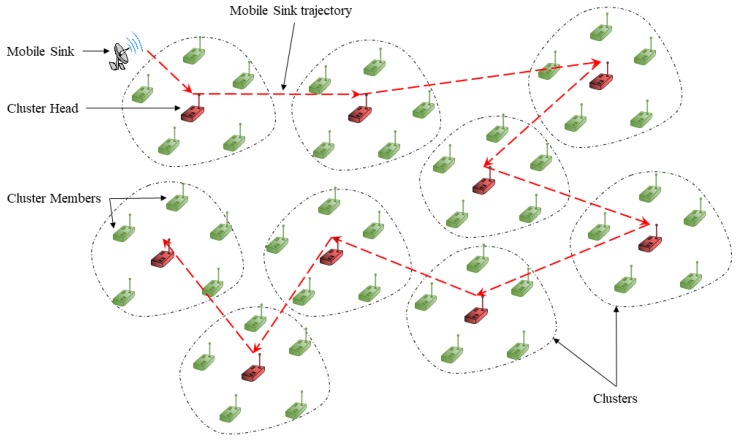
Moving plan in Cluster-Based Wireless Sensor Network.

The path passed by the MS in the wireless sensor network is an essential and influencing factor in the data collection process. Hence, we need to determine the mobility model to implement it in the MS. To do so, we base on the mobility model used in a previous publication [15], in which the MS passes by all sensor nodes. Thus, we adopt this model mobility to cluster-based wireless sensor network in order to make MS passes only by CHs in the network field. Figure 2 shows an example of how the MS moves through the network.

Finding the moving plan for the MS passing by all CHs in the network is similar to the well-known traveling salesman problem (TSP). TSP is considered an NP-hard problem. Thus, the problem of finding the shortest path for the MS is an NP-hard problem, where the MS acts as salesman and CHs are the cites to be visited by the MS [15]. We assume that the MS knows the topology of the network and it calculates the shortest path passing by all CHs in the cluster-based wireless sensor network. The authors in [15], used mobility models based on metaheuristic algorithms. Metaheuristic algorithms are optimization methods used for solving NP-hard class problems. These methods search solutions for problems in reasonable calculation time. These solutions may be optimum solutions or very close to the optimum solutions. 

We have chosen a mobility model from the previous publication [15] based on a tabu search algorithm, and the authors used a multi-objective function with different constraints to solve the problem of finding a suitable trajectory for MS. In this work, we are interested in finding the shortest path for the MS based on distance. Therefore, we only use the part of calculating the shortest trajectory of the multi-objective proposed in [15]. The objective function used in this work is presented in Equation (2) which minimizes the path of MS in terms of distance. The metaheuristic tabu search method used in this paper for calculating the trajectory is presented in Algorithms 1 and 2.

*Problem Statement.* The metaheuristic algorithm used to calculate the optimal shortest path passing by all CHs in order to collect data by taking into consideration some related constraints.

### 4.1. Optimization Model

We present in the following the optimization model used to obtain the optimal path trajectory of the MS. The function *f* in Equation (2) represent the objective function which minimizes the tour length of the MS in term of distance:

(2) $$f:\min\;\overset{n}{\underset{i,j\in C,i\neq j}{\sum }}c_{ij}d_{ij}$$

Subject to:

(*i*) $$0\leq d_{ij}\leq 1\;\forall i,j\in C;\;i,j=1,\ldots ,n;\;i\neq j;$$

(*ii*) $$\sum_{i=1}^{n}d_{ij}=1;\;\forall i,j\in C;\;i\neq j;$$

(*iii*) $$\sum_{j=1}^{n}d_{ij}=1;\;\forall i,j\in C;\;j\neq i;$$

(*iv*) $$\sum^{n}d_{ij}\leq n-1;\;\forall i,j\in C;i\neq j;$$

(*v*) $$E_{i}\geq E_{min};\;\forall i\in C;$$

where *C* designates the set containing n cluster heads in the wireless sensor network, $c_{ij}$ represents the distance between two cluster head nodes (the distance between cluster head i and cluster head j). *d_ij_* is a binary value (0 or 1) which indicates whether there is a displacement from cluster head *i* to cluster head j in the optimal path or trajectory which is defined in constraint (i). 

Constraint (ii) makes the MS visits only one CH j after it visits a CH i. The MS visits a CH j and it must come from only one CH *i* which is expressed in constraint (iii). Constraint (iv) ensures that the tour of the MS is fully connected, and it visits all CHs in the network. Constraint (v) is defined to ensure that the CH to be visited have enough energy to transmit data toward the MS.

In Equation (1), $c_{ij}$ is calculated by Equation (3):

(3) $$c_{ij}=\sqrt{{\left(w-u\right)}^{2}+{\left(z-v\right)}^{2}}\;\forall i,j\in C,i\neq j.\;\forall \left(w,z\right),\left(u,v\right)\in R^{2}$$

where (w,z) and (u,v) are the coordinates of the cluster head i and j respectively in two dimensions zone (2D).

In Equation (2), $d_{ij}=\{\begin{array}{c} 1,\;\mathrm{If}\;\mathrm{there}\;\mathrm{is}\;a\;\mathrm{displacement}\;\mathrm{from}\;i\;\mathrm{to}\;j\;\mathrm{in}\;\mathrm{the}\;\mathrm{optimal}\;\mathrm{path}.\\ 0,\;\mathrm{else}. \end{array}$

### 4.2. Optimization Method

The tabu search algorithm is considered one of metaheuristic algorithms used to find optimal solutions for problems. This method is also used to solve the TSP problem, which is similar to the problem of finding the moving plan of the MS [30,31]. Tabu search is based on a local search procedure to search for an improved solution from a potential solution using a neighborhood search function and tabu list to avoid dealing with solutions already obtained. The search for an optimal solution continues until a stop condition is reached. 

The role of Algorithm 1 is to find the best neighbor of a solution in the search space. In line 3 to 12, the algorithm searches the best cost in terms of distance of a solution by the permutation in the order of cluster heads in the itinerary of the MS (lines 5 and 6). Then it calculates the cost of the neighbor of the current solution (line 7). If the cost of this neighbor solution is better than the previous one and it is not in the tabu list (line 8), then this neighbor solution is selected as the best neighbor of the current solution (lines 9 and 10). After searching with all cluster heads, the best solution is returned as the best neighbor solution (line 14).

**Algorithm 1:** Neighborhood Search**1** $Sol^{\prime }=Sol$; /*Initiate the $Sol^{\prime }$ with the initial solution Sol .*/**2** $BestCost=f\left(Sol^{\prime }\right);$ /* To calculate current cost*/**3 *For (i = 1 To N) Do*** /*N is the number of Clusters*/**4**    ***For (j = 1 To N) Do*****5**      $TempSol=Sol^{\prime }$; /* TempSol: Temporary Solution*/**6**      $TempSol=\mathrm{Permutation}\left(CH_{i},CH_{j}\right)$; /* Switching between Cluster Heads */**7**      BestTempCost=f(TempSol); /*The cost of TempSol */**8      *If****(*BestTempCost<BestCost*)**And**(*TempSol≠T*)****Then*** /* T: Tabu List*/**9**          $Sol^{\prime }=TempSol$ ;**10**        BestCost=BestTempCost ;**11**      ***EndIf*****12**    ***EndFor*****13** ***EndFor***
**14 *Return***
$Sol^{\prime }$


Algorithm 2 is the main algorithm of tabu search. This algorithm is based on a tabu list to avoid dealing with the previous solution (line 3) and the number of the maximal number of iterations to search the best solution (line 4). First, an initial solution must be defined to process the Tabu search (line 1 and 2). While the number of iterations is still less than the maximal iteration number (line 5 to 13), the search of the best path for the MS passing by all CHs in the network is processing. In this search, the algorithm searches the best neighbor of the current solution, which is not tabu using Algorithm 1 (lines 6 to 10). At each iteration, the tabu list is updated (line 11). When the number of iterations reaches the maximal number, the algorithm returns the best solution found (line 14).

**Algorithm 2** Tabu Search algorithm for the Mobile Sink**1** Sol=IT; /*Initial Solution: Initial Trajectory (IT) */**2** $f^{*}=f\left(Sol\right)$; /*Calculate the cost of the solution Sol using f (Objective Function) */**3** T=∅; /* Initiate the tabu list */**4** I=0; $I_{max}=i_{max}$; /* $i_{max}$: The maximal number of iteration */
**5 *Repeat***
**6**   $Sol^{\prime }=$ The best neighbor of the solution *Sol* is not tabu (Algorithm 1);**7       *If*** $f\left(Sol^{\prime }\right)<f\left(Sol\right)$ ***Then*****8**         $Sol=Sol^{\prime }$;**9**         $f^{*}=f\left(Sol\right)$;**10**      ***EndIf*****11**  Update the tabu list T;**12**  *I++*; ***13 Until (Stop Condition***$\left(I=I_{max}\right)$***)***;**14 *Return***Sol.

## 5. Designed Protocols

In this section, we show our main contributions in this paper, which are the creation of our proposed protocol CLR-MSPH and the adaptation of BMAC protocol with handover process (BMAC-H) in order to compare the performance of the proposed protocol CLR-MSPH to BMAC-H protocol. We present both CLR-MSPH and BMAC-H, with their design and their different concepts in details. We also discuss the problem of the collision during the data transfer process.

### 5.1. Adapted BMAC Protocol with Handover Process for Cluster-Based Wireless Sensor Network (BMAC-H)

**Figure 3:**
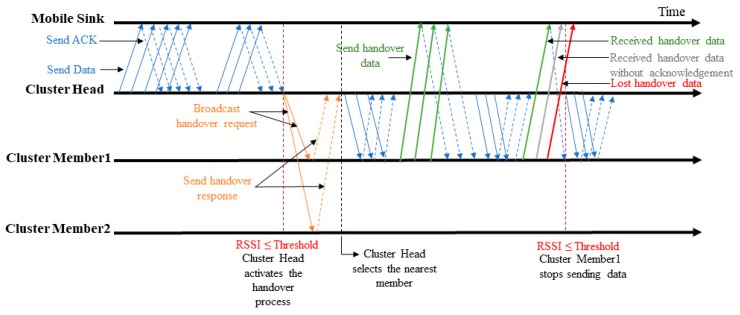
BMAC-H behavior.

In this subsection, we adapt the BMAC protocol with the handover mechanism to cluster-based wireless sensor networks in order to compare it to the proposed protocol CLR-MSPH. Figure 3 illustrates the behavior of the CH when the MS approaches to leave the radio range of CH. Algorithm 3 shows the different instructions executed in a CH to perform the handover.

For the detection of MS movements, we use the ACK frame to measure the RSSI value. We set up the threshold for RSSI value to −90 dBm (line 4, Algorithm 3). When CH receives an ACK from the MS after a data sent with an RSSI value equals or less than the RSSI threshold. Based on this value, the CH detects the degradation in RSSI value, and then it starts the handover process by broadcasting a HANDOVER_REQUEST to its members (line 11, Algorithm 3). CM, who receives the HANDOVER_REQUEST, sends a HANDOVER_RESPONSE to CH. CH sets a timer to wait for possible HANDOVER_RESPONSE from CMs (line 12 to 15, Algorithm 3). After this, the CH calculates the closest CM to select it as a relay in order to continue transferring data to the MS (line 16, Algorithm 3). The selected CM receives data from CH and forwards it to the MS while the latter passing through the radio range of this relay CM. However, BMAC-H works on the MAC Layer, and the maximum number of hops for route data is 2 hops, i.e., CH sends data packets to CM then CM forwards them to MS.

**Algorithm 3** BMAC-H algorithm at CH for Cluster-Based Wireless Sensor Network**1** SendDataPacket (***MS@***);**2** SetTimer (WaitForACK_DATA, *timer1*);**3 *If*** (ACK_DATA *received*) ***Then*****4   *If*** (RSSI (ACK_DATA) > *Threshold*) ***Then*****5    *If*** (Buffer_Size() > 0) ***Then*****6**       GoTo 1;**7**    ***Else*****8**       Goto 33;**9**    ***EndIf*****10**   ***Else*****11**    Broadcast (HANDOVER_REQUEST);**12**    SetTimer (WaitForHANDOVER_RESPONSE, *timer2*);**13    *While*** (HANDOVER_RESPONSE *received*) ***And*** (*timer2* > 0) ***Do*****14**      CandidateCMForHandover.add (***CM***);**15**    ***EndWhile*****16**    Select the nearest ***CM***;**17**    SendDataPacket (***CM@***);**18**    SetTimer (WaitForACK_DATA, *timer1*);**19    *If*** (ACK_DATA *received*) ***Then*****20      *If*** (Buffer_Size() > 0 ) ***Then*****21**       GoTo 17;
**22      *Else***
**23**       GoTo 33;**24**      ***EndIf*****25**    ***Else*****26**      RetransmissionDataPacket (***CM@***);**27**      GoTo 17;**28**    ***EndIf*****29**   ***EndIf*****30** ***Else*****31**  RetransmissionDataPacket (***MS@***);**32**    GoTo 2;**33** ***EndIf***

### 5.2. Cross-Layer Routing for Mobility Support Protocol Based on Handover Process (CLR-MSPH)

In this subsection, we present our proposed CLR-MSPH, which is based originally on the BMAC protocol. CLR-MSPH is considered as an extending implementation of BMAC-H by integrating the prediction of relay nodes, multihop routing of data, controlling the handover mechanism, and involving uplevel layer (network layer) for better routing of data. 

CLR-MSPH protocol performs mainly on two layers, which are network layer and MAC layer in addition to the mobility management plan. The two layers exchange information in order to have better knowledge about the network and reduce the signaling costs between nodes so that the energy consumption of wireless sensor network. We also present in this subsection, the detailed design of CLR-MSPH, such as how to predict and select relay nodes. We also show the different stages of the CLR-MSPH performing to select these relay nodes.

**Figure 4:**
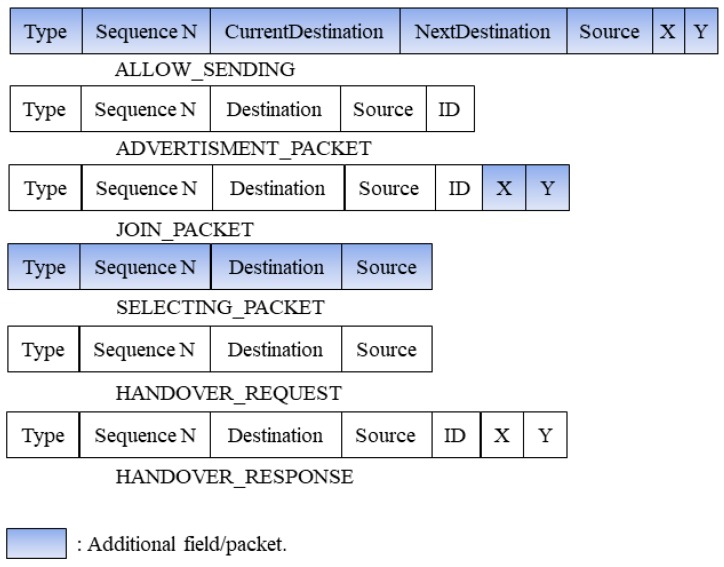
The different Packets used in CLR-MSPH.

In the phase of forming clusters, we are only interested in the ADVERTISEMENT_PACKET and the JOIN_PACKET (Figure 4). In this phase, the candidate CHs broadcast an advertisement packet to offer other nodes to join their clusters. The node which accepts the offer replies by JOIN_PACKET to inform the designated CH that it joins to the cluster. We add to JOIN_PACKET some information fields such as the joining node’s localization and its ID. This information is needed by CH to calculate the distance between its members and to estimate the handover node (HN) to route data in the case of the MS leaves the radio range of CH without sending all data.

Figure 4 illustrates the essential packets used in the proposed CLR-MSPH protocol, where we modified some packets by adding some mandatory fields for the proposed protocol. We also created the SELECTING_PACKET, and we attributed to it the necessary fields for the CLR-MSPH. The information in these packets helps in predicting and selecting the handover node (HN).

**Figure 5:**
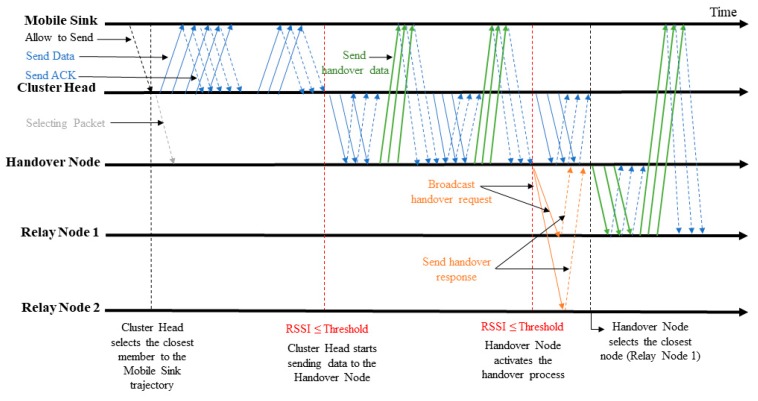
The behavior of the proposed CLR-MSPH.

Figure 1 and Figure 5 and Algorithm 4 show the behavior of our proposed protocol CLR-MSPH. The MS broadcasts a HELLO message periodically and waits for an ACK_HELLO message from CHs. A CH has ready data in its buffer to transfer to the MS is a candidate CH for sending data. Only candidate CH will reply by ACK_HELLO message to the MS. When the MS receives the acknowledgment of HELLO message from a CH, it sends ALLOW_SENDING packet to this CH in order to make the CH starts transferring data from its buffer (line 1, Algorithm 4). The MS integrates into the ALLOW_SENDING packet its next destination after the current CH with coordination. The next destination filed in this packet, allows the current CH to predict and select the suitable CM to forward data in the case of the CH estimates that it cannot send all the data in its buffer (line 2, Algorithm 4). If it is the case, CH sends a SELECTING_PACKET to this selected node called handover node (HN) to inform it that it is selected to forward data to MS (line 3, Algorithm 4). 

When a CM receives a SELECTING_PACKET, i.e., it becomes an HN, this HN processes the same as CH when the latter receives a HELLO message from the MS. This SELECTING_PACKET packet makes this HN acknowledges the periodic HELLO message sent by MS in order to receive the ALLOWING_SENDING packet to activate the transfer of data to the MS. 

**Algorithm 4** CLR-MSPH algorithm at CH for Cluster-Based Wireless Sensor Network**1 *If*** (ALLOW_SENDING *received*) ***Then*****2**   Estimate the HN using the fields in ALLOW_SENDING packet;**3**   SendSelectingPacket (***HN@***);**4**   SendDataPacket (***MS@***);**5**   SetTimer (WaitForACK_DATA, *timer1*);**6   *If*** (ACK_DATA *received*) ***Then*****7     *If*** (RSSI (ACK_DATA) > *Threshold*) ***Then*****8**       ***If*** (Buffer_Size() > 0) ***Then*****9**         GoTo 4;**10**       ***Else*****11**         Goto 31;**12**       ***EndIf*****13**     ***Else*****14**       SendDataPacket (***HN@***);**15**       SetTimer (WaitForACK_DATA, *timer1*);**16**       ***If***(ACK_DATA *received*) *Then***17         *If*** (Buffer_Size() >0 ) ***Then*****18**          GoTo 14;
**19         *Else***
**20**          GoTo 31;**21**         ***EndIf*****22**       ***Else*****23**         RetransmissionDataPacket (***HN@***);**24**         GoTo 14;**25**       ***EndIf*****26**     ***EndIf*****27**   ***Else*****28**    RetransmissionDataPacket (***MS@***);**29**    GoTo 4;**30**   ***EndIf*****31** ***EndIf***

As in BMAC-H, the handover process of data starts when the last ACK frame received from MS with RSSI value less or equal to the RSSI threshold. At this moment, the CH sends data directly to HN, which is already selected by CH to handover data to MS (line 14, Algorithm 4). In the case where CH estimates that it cannot finish sending all data in its buffer and the MS starts leaving its radio range, CH sends the remaining data directly to the HN then HN routes them to MS in two hop routing. 

Predicting and preselecting the HN occurs in the phase of allowing the MS to CH to send data by sending the ALLOW_SENDING packet. This phase allows reducing the handover delay compared to BMAC-H, in which CH launches the search of the relay node when the MS is about to leave the radio range of CH. We discuss in detail the phase of predicting the HN further below.

In some cases, MS may leave the radio range of the HN without transferring all the handover data from CH due to a large amount of data. The proposed CLR-MSPH deals with this problem by allowing HN to process the handover mechanism. In this case, HN processes a similar handover to the BMAC-H (Algorithm 3), where HN broadcasts a HANDOVER_REQUEST, and it sets a timer to receives all possible HANDOVER_RESPONSE from other nodes in order to select the suitable relay node to process handover data. By allowing HN processes the handover mechanism, CLR-MSPH may perform in three hop routing.

The communication between the MS and nodes keep till the RSSI value is −93 dBm (tested in the Omnet++/Castalia [32] simulator; the last ACK received is with a −93 dBm RSSI value). In this work, we set the threshold to −90 dBm to perform the handover process. Therefore, we introduce a controlling mechanism for transmitting stored data in the buffer to MS (Algorithm 5). We make the CH and HN check their buffers while they are sending data to MS and receiving acknowledgments (ACK) of data from the MS (line 1, Algorithm 5). If the RSSI value of ACK message is restricted between −90 dBm and −92 dBm (line 2, Algorithm 5) and the size of the buffer is less than PDR (the default packets delivery rate is 5 pcks/sec), CH or HN continues sending data to MS without performing handover (line 3, Algorithm 5). We believe that it is not necessary to handover a small amount of data in the buffer while it is convenient and enough to send them in this interval of RSSI values. In addition, sending data using this controlling mechanism will reduce the latency (end-to-end delay), avoid the risk, and reduce the probability of losing data packets during the handover process. In the other hand, when the buffer of the CH has a large amount of data, CH performs the handover and transfers data to its HN (line 6, Algorithm 5). The same with the HN, where it performs the handover with the node which is selected by HN (line 8, Algorithm 5).

**Algorithm 5** Control mechanism for transmitting data from buffer of CLR-MSPH**1 *If*** (Buffer_Size() ≤ ***PDR***) ***Then*****2**     ***If** (**RSSI*** < -90*) **And** (**RSSI*** > -92*) **Then*****3**       Packet.setDestination(***MS@***);
**4     *Else***
**5       *If*** (***isCH***) ***Then*****6**         Packet.setDestination(***HN@***);**7**       ***If** (**isHN**) **Then*****8**          Packet.setDestination(***Node@***); /*Node is selected by HN to handover data*/**9**     ***EndIf*****10** ***EndIf***

#### 5.2.1. Predicting Handover Node (HN)

The CH receives an ALLOW_SENDING packet with the next destination of the MS. This information helps the CH to predict the HN and to select it for routing the data in the case of CH does not finish transfer all the data in its buffer while the MS passes through its radio range. The CH already knows all the positions of its members thanks to the integration of this information into the JOIN_PACKET in the phase of forming clusters. Where each CM integrates into JOIN_PACKE, its ID and localization then it sends the joining packet to CH (Figure 4).

To select the Handover Node (HN), CH performs the following:

(1) Find all cluster members (CMs) which are in the same direction as the mobile sink (MS).
(2) Calculate the distance between the CM (which are in the same direction as the MS) and the trajectory line of the MS.
(3) Search for the closest CM to the trajectory line of the MS and select it as HN by sending the SELECTING_PACKET.

(1) CMs in the same direction as MS

**Figure 6:**
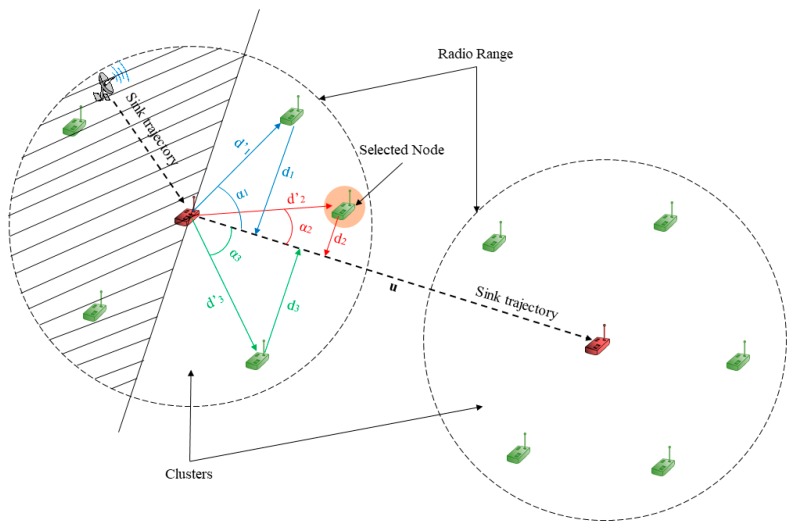
Selecting the closest CM to the trajectory of the sink.

First of all, CH finds all the CMs who are in the same direction as the MS trajectory. To do so, CH calculates the cosine of the angle between the vector from $CH_{i}$ to $CM_{k}$ (vector $\vec{{d^{\prime }}_{k}}$) and the vector from the current $CH_{i}$ to the next $CH_{j}$ (vector $\vec{u}$, see Figure 6). If the cosine angle is greater than 0, then the $CM_{k}$ is in the same direction of MS trajectory. Otherwise, the $CM_{k}$ is not in the same direction of the MS trajectory.

The cosine angle between the vectors $\vec{u}$ and $\vec{{d^{\prime }}_{k}}$ in Equation (4):

(4) $$\cos\alpha =\frac{\vec{u}\cdot \vec{{d^{\prime }}_{k}}}{\left|\left|\vec{u}\right|\right|\cdot \left|\left|\vec{{d^{\prime }}_{k}}\right|\right|}$$

where $\vec{u}=\left(\begin{array}{c} x_{CH_{j}}-x_{CH_{i}}\\ y_{CH_{j}}-y_{CH_{i}} \end{array}\right)$, $\vec{{d^{\prime }}_{k}}=\left(\begin{array}{c} x_{CM_{k}}-x_{CH_{i}}\\ y_{CM_{k}}-y_{CH_{i}} \end{array}\right)$ and $\vec{u}\cdot \vec{{d^{\prime }}_{k}}$ is the dot product or scalar product of vectors $\vec{u}$ and $\vec{{d^{\prime }}_{k}}$.

$\left|\left|\vec{u}\right|\right|$ and $\left|\left|\vec{{d^{\prime }}_{k}}\right|\right|$ are the length of vectors $\vec{u}$ and $\vec{{d^{\prime }}_{k}}$ in Equations (5) and (6):

where:

(5) $$\left|\left|\vec{u}\right|\right|=\sqrt{{\left(x_{CH_{j}}-x_{CH_{i}}\right)}^{2}+{\left(y_{CH_{j}}-y_{CH_{i}}\right)}^{2}}$$

(6) $$\left|\left|\vec{{d^{\prime }}_{k}}\right|\right|=\sqrt{{\left(x_{CM_{k}}-x_{CH_{i}}\right)}^{2}+{\left(y_{CM_{k}}-y_{CH_{i}}\right)}^{2}}$$

The result of Equation (4):

$$\cos\alpha \{\begin{array}{cc} >0, & the\;CM\;is\;in\;the\;same\;direction\;as\;the\;MS.\\ \leq 0, & the\;CM\;is\;not\;in\;the\;same\;direction\;as\;the\;MS. \end{array}$$

(2) Distance between CMs and MS’s trajectory line

After selecting all the CMs which are in the same direction of the sink trajectory, CH finds the closest CM to the MS trajectory line. To find the distance between $CM_{k}$. and the MS trajectory line, we need to find the line equation of the trajectory then calculate the distance between CMs and trajectory line. CH determines the equation of the MS trajectory using its coordinates (current CH coordinates) and the coordinates of the next destination (next CH coordinates to visit, CH gets this information from the ALLOW_SENDING packet).

The trajectory line equation of the MS from a cluster head i ($CH_{i}$) to a cluster head j ($CH_{j}$) is defined in Equation (7):

(7) $$y=m_{ij}x+b_{ij}$$

where $m_{ij}$ is the slope between $CH_{i}\left(x_{CH_{i}},y_{CH_{i}}\right)$ and $CH_{j}\left(x_{CH_{j}},y_{CH_{j}}\right)$. $m_{ij}$ is calculated in Equation (8):

(8) $$m_{ij}=\frac{y_{CH_{j}}-y_{CH_{i}}}{x_{CH_{j}}-x_{CH_{i}}}$$

In some cases, the two cluster heads $CH_{i}$ and $CH_{j}$ have the same x coordinates, i.e., $x_{CH_{j}}-x_{CH_{i}}=0$ then $m_{ij}$ is undefined. In this case, Equation (7) becomes as follows:

$$\{\begin{array}{c} y=m_{ij}x+b_{ij},\;if\;x_{CH_{j}}\neq x_{CH_{i}}\\ x=x_{CH_{j}}\;or\;x=x_{CH_{i}},\;if\;x_{CH_{j}}=x_{CH_{i}} \end{array}$$

$b_{ij}$ is determined after $m_{ij}$ is calculated in Equation (7) and using the coordinates of $CH_{i}$. $b_{ij}$ is calculated in Equation (9):

(9) $$b_{ij}=y_{CH_{i}}-m_{ij}x_{CH_{i}}$$

The distance $d_{k}$ is the distance from a $CM_{k}$ to the trajectory line of the MS. This distance is calculated in Equation (10):

(10) $$d_{k}=\frac{\left|ax_{CM_{k}}+by_{CM_{k}}+c\right|}{\sqrt{a^{2}+b^{2}}}$$

where:

(11) $$a=m_{ij}^{-1}m_{ij}$$

(12) $$b=-m_{ij}^{-1}$$

Also:

(13) $$c=m_{ij}^{-1}b_{ij}$$

With $m_{ij}^{-1}$ in Equation (11), is the inverse of $m_{ij}$ thus, $m_{ij}^{-1}$ is calculated from Equation (8) in Equation (14):

(14) $$m_{ij}^{-1}=\frac{x_{CH_{j}}-x_{CH_{i}}}{y_{CH_{j}}-y_{CH_{i}}}$$

Therefore a in Equation (11) is, a=1.

Equation (10) becomes as follows:

(15) $$d_{k}=\frac{\left|x_{CM_{k}}-m_{ij}^{-1}y_{CM_{k}}+m_{ij}^{-1}b_{ij}\right|}{\sqrt{1^{2}+{\left(-m_{ij}^{-1}\right)}^{2}}}$$

$d_{k}$ in Equation (15) represents the distance between a $CM_{k}$ and the trajectory line of the MS.

(3) The closest CM to the MS’s trajectory is HN

The CH searches the closest CM to the trajectory line and on the same direction of the MS path. CH selects this CM to be the HN by sending the SELECTING_PACKET, then routes the data to the selected HN in the case of MS leaves the communication area before sending all data stored in the buffer (Figure 6).

#### 5.2.2. The Layered Architecture of CLR-MSPH

**Figure 7:**
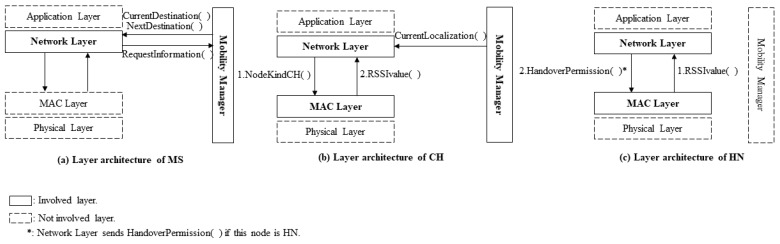
Involved layers in CLR-MSPH. (**a**) The layered architecture of the MS; (**b**) The layered architecture of a CH; (**c**) Layered architecture of an HN.

In this part, we show the layered architecture used in our proposed protocol CLR-MSPH. Figure 7 illustrates the different layers involved and cooperated in each kind of node in the Cluster-based wireless sensor network. We involve the network layer to provide efficient mobility support for our proposed CLR-MSPH because we believe that this layer handles the mobility better than other layers. The main reason to involve the network layer is that this layer uses the information coming from the mobility manager to estimate and predict the appropriate node for performing the handover and routing the data.

Mobility management allows the network layer to get information about localization, which is used for calculating distances. Figure 7a shows the interactions of layers in the MS, the network layer sends a direct control message to mobility management to request the current destination and the next destination with their coordinates. The network layer uses this information coming from mobility management to encapsulate it in the ALLOW_SENDING packet. MS sends this packet to current CH (current destination) in order to make this CH start sending data and predicts the HN.

Figure 7b illustrates the layers involved in the CH. As we mentioned before, the network layer handles the mobility better than other layers. The communication between this layer and the mobility management is used to estimate and calculate the HN. For making the handover perform efficiently and for better knowledge about the network, we also make MAC layer, and network layer cooperate. The network layer informs the MAC layer that the type of this node is CH by sending a control message to the MAC layer in order to make this node interacts in the case of handover mechanism. When an ACK frame is received at the MAC layer of a CH with a RSSI value less or equal to the RSSI threshold, the MAC layer sends a control message to network layer to inform it that the RSSI reaches the threshold. Depending on this control message, the network layer changes the destination of packets from the sink address to the preselected HN address to handover the remaining data.

Figure 7c shows the layered architecture of the HN. The network layer and MAC layer also cooperate for better handover. CH selects a node as HN by sending SELECTING_PACKET which is created at the network layer of CH, and it is received at the network layer of the receiving node (HN) to inform it that it is selected as HN. In the case where an ACK frame is received from MS with RSSI value less or equal to the threshold, the MAC layer sends a control message to the network layer to get permission to process the handover mechanism at the MAC layer of the HN (process the similar handover of BMAC-H). Once the MAC layer gets the information and the permission to process handover, HN creates at the MAC layer a HANDOVER_REQUEST frame and broadcasts it, and then it waits for HANDOVER_RESPONSE from other nodes.

#### 5.2.3. Collision Problem

Collision problems are a common issue is wireless communications. This problem is caused by the simultaneous sending of packets from source nodes to the same destination node. Different solutions are proposed in the literature, among which we can cite the TDMA and RTS/CTS mechanisms which dealt with collision problems.

The collision problem is also considered in this paper. While the MS is moving among clusters, a collision of packets may occur during the process of data collection phase or handover process phase. The collision problem is due to the simultaneous transmission of packets from two or more source nodes (CHs) to the same destination node (MS). This problem affects the performance of data collection, and it degrades the reception rate of packets. Therefore, to enhance the data collection process and reduce the probability of collisions, we propose a simple mechanism for this problem in which we set a random backoff delay or timer for each source node in order to avoid the simultaneous sent, where each source node has a different time of sending. 

## 6. Simulations and Results

We test our proposed protocol CLR-MSPH and BMAC-H using the Simulator Omnet++/Castalia [32], which is a powerful and flexible simulator, and it supports the mobility of nodes. We simulate both protocols by taking into consideration the variation of speed because the velocity of the MS affects the transfer of data and the handover mechanism in each protocol. We also simulate CLR-MSPH and BMAC-H on different and random topologies. Table 1 shows the different parameters of the simulations.

### 6.1. Scenario 1

In scenario 1, we test the network performances of both protocols CLR-MSPH and BMAC-H with varying the speed of the MS in a random topology of 60 sensor nodes with 10 clusters in addition to the MS. We compare the results of simulation in terms of packet reception rate (PRR), latency (end-to-end delay) and energy.

#### 6.1.1. Packets Reception Rate

**Figure 8:**
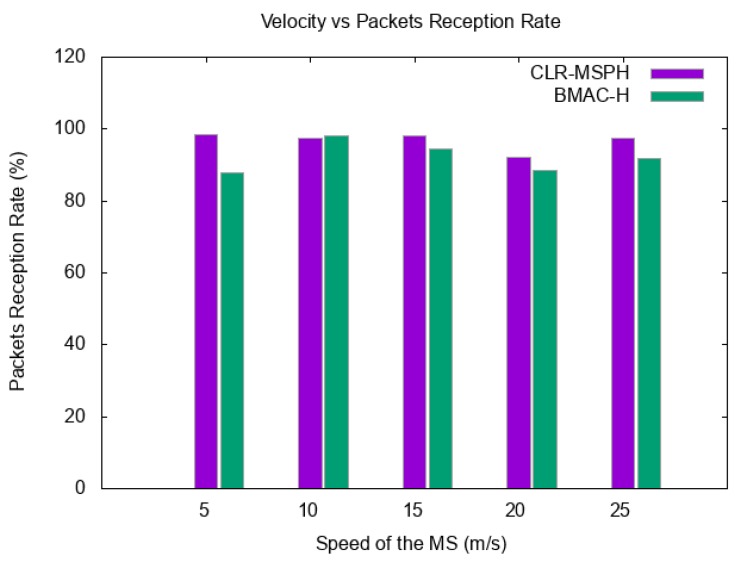
Packets Reception Rate VS the velocity of the MS in m/s.

**Figure 9:**
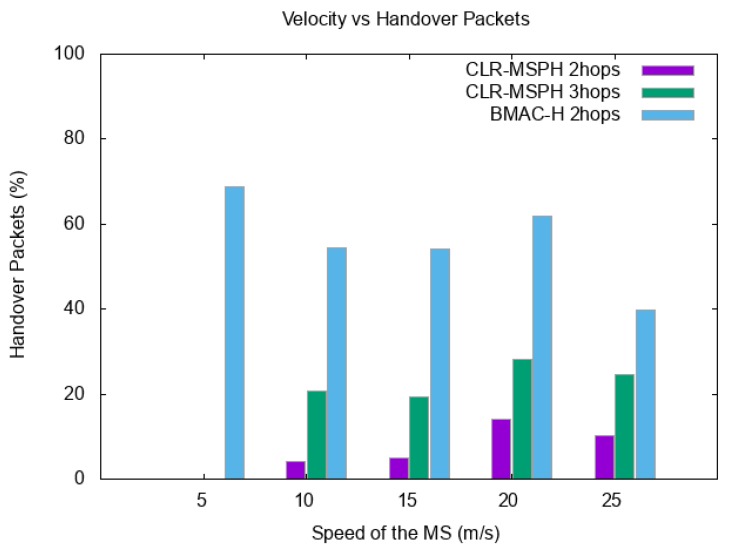
Handover packets in each protocol with varying the speed of MS.

In this part, we discuss the packets reception rate (PRR). In order to study the control of the handover mechanism of each protocol, we also study the percentage of delivered packets using the handover mechanism in each protocol and the number of hops to route these packets to the MS. Figure 8 and Table 2 represent the total PRR of both protocols with varying the speed of the MS. In the other hand, Figure 9 and Table 3 illustrate the impact of the MS velocity on the handover mechanism for both CLR-MSPH and BMAC-H protocols.

We notice that our proposed CLR-MSPH performs better than the BMAC-H protocol in terms of PRR in almost all the cases with varying the velocity of the MS from 5 m/s to 25 m/s. The CLR-MSPH gives the greatest PRR of 98.46% and the lowest PRR of 92.17%. On the other hand, BMAC-H protocol performs less than our proposed CLR-MSPH protocol, where the greatest PRR picks at 98.21% and significantly falls to the lowest PRR at 87.83% (Figure 8 and Table 2).

We also see for the packets received at the MS using both protocols that CLR-MSPH has more control of the handover mechanism, where it shows the lowest percentage of packet delivered using handover thanks to the control mechanism of CLR-MSPH in Algorithm 5. This control of handover helps in reducing the packet loss probability, and that is confirmed in the results in terms of PRR in which CLR-MSPH has the highest PRR compared to BMAC-H in almost all cases with varying the speed of MS. The check of buffer size mechanism explained before in CLR-MSPH helps in controlling the handover and reducing the number of packets sent using handover to avoid packets loss. We also see that the handover mechanism used in CLR-MSPH performs in three hop routing compared to handover mechanism used in BMAC-H which performs in two hop routing.

In the case where the speed of the MS is 5 m/s, the CLR-MSPH did not process the handover because the speed of MS is sufficient to deliver data from the CH to the MS without processing the handover with the help of the controlling handover mechanism in CLR-MSPH, while BMAC-H handovers 68.68% of data received at the MS in the simulation, which explains the lowest PRR of 87.83% (Figure 9 and Table 3). 

#### 6.1.2. Latency (End-to-End Delay)

Usually, the latency in mobile wireless sensor networks refers to the time necessary for sending a data packet through the network from a source node which is a sensor node to the final destination which is the MS. The latency in cluster-based wireless sensor networks with mobile sinks used in Omnet++/Castalia is calculated from the time *T_s_* when a packet is received from a CM at CH and stored in the CH’s buffer or some cases, the CH generates packets in its application layer, in this case, $T_{s}$ will be the time when the CH stores its own data in the buffer, to the time $T_{r}$ when the packet is received at MS.

The latency of a packet $L_{pi}$ in Omnet++/Castalia is calculated as follows using Equation (16):

(16) $$L_{pi}=T_{ri}-T_{si}$$

The average latency L of all packets received by the mobile sink is calculated by Equation (17):

(17) $$L=\frac{1}{N_{p}}\overset{N_{p}}{\underset{i=1}{\sum }}L_{pi}$$

where *N_p_* is the number of received packets.

Table 4 shows the end-to-end delay of packets received at the MS with varying its velocity using both CLR-MSPH protocols. We notice that there is a slight difference in the latency between CLR-MSPH and BMAC-H. The latency of packets using CLR-MSPH is slightly higher than the latency of packets using BMAC-H. This slight difference is due to the number of hops used for routing the data in CLR-MSPH compared to BMAC-H, i.e., the increase of the number of hops increases the latency of packets. In case of MS’s speed is 5m/s the CLR-MSPH has a latency less than BMAC-H because CLR-MSPH did not perform the handover process and the CHs sent data directly to the MS, i.e., the speed of MS is enough to send all the data packets in one single hop from CH (Table 4).

#### 6.1.3. Energy

**Figure 10:**
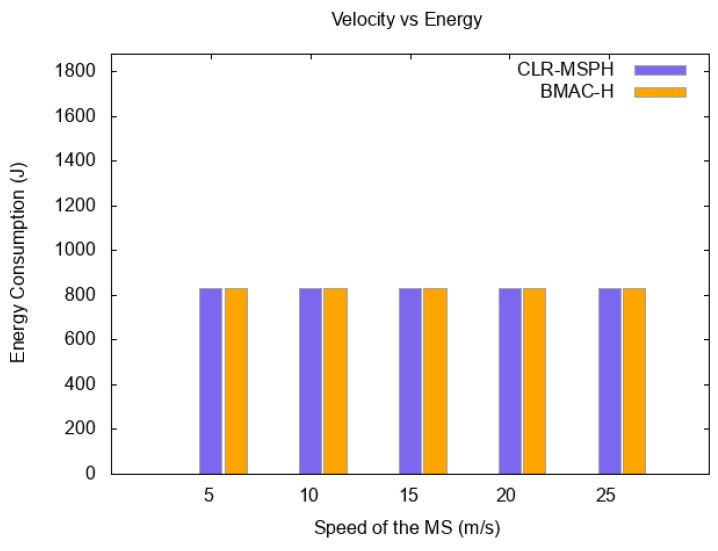
Energy consumed in each protocol with varying the speed of the MS.

In wireless sensor networks the energy is considered one of the most critical constraints. The hierarchical architecture of the network is widely used as a solution to save energy and organize the network, especially in large-scale networks. Figure 10 illustrates the energy consumed using the handover mechanisms of both CLR-MSPH and BMAC-H when the speed of the MS is varied. The energy consumed by BMAC-H is slightly more than the energy consumed by CLR-MSPH in all cases, while the speed of the MS is varying (Figure 10).

### 6.2. Scenario 2 

In this scenario, we study the scalability of both protocols CLR-MSPH and BMAC-H with different random topologies to see the efficiency of our proposed CLR-MSPH protocol. We test both protocols on topologies with 30, 60, 90, 120 sensor nodes and when the average number of clusters in each topology is 5, 10, 15, 20, respectively, in addition to the sink in a random deployment of nodes with a 15 m/s default speed of the MS. 

**Figure 11:**
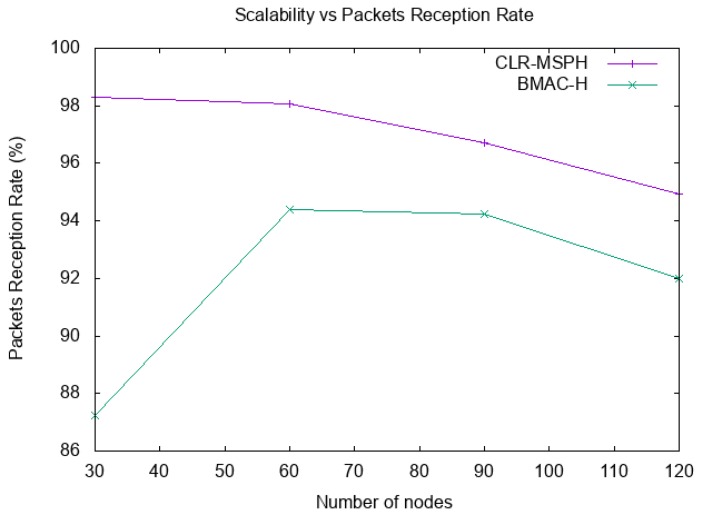
Scalability and Packets Reception Rate in each protocol.

**Figure 12:**
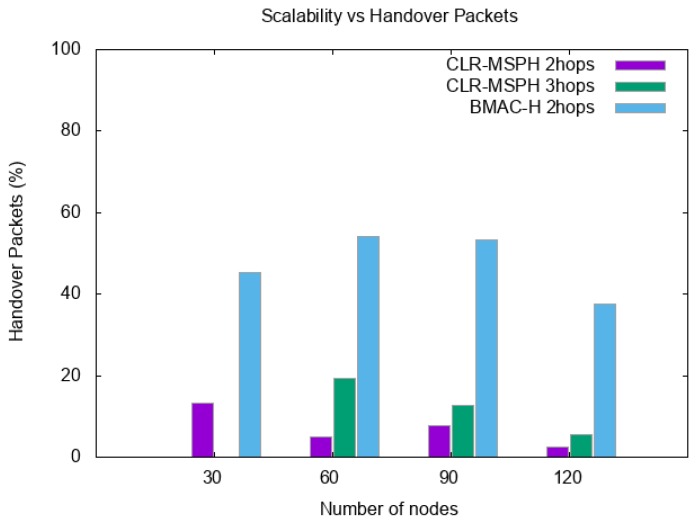
Scalability and percentage of handover data in each protocol.

**Figure 13:**
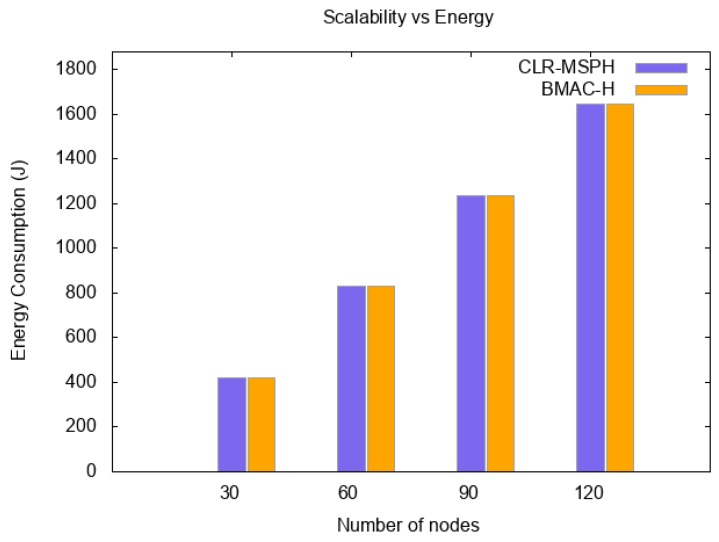
Energy consumption of both protocols in each topology.

We study the scalability of the proposed CLR-MSPH protocol and BMAC-H protocol in terms of packet reception rate (PRR), latency, and energy consumption. Figure 11 and Figure 12 and Table 5 and Table 6, present the total PRR and the percentage of data delivered using the handover mechanisms of both CLR-MSPH and BMAC-H when the number of nodes in the network is varying. The latency of data delivered at the MS is presented in Table 7, and Figure 13 illustrates the energy consumed in each topology using both the CLR-MSPH and BMAC-H protocols.

We notice that our proposed CLR-MSPH protocol performs better than the BMAC-H protocol in all cases in terms of PRR, latency, and energy. In terms of latency (end-to-end delay), we notice that CLR-MSPH performs better than BMAC-H, where our proposed protocol delivers packets with the shortest end-to-end delay. In the case of 30 nodes in the network, we notice that CLR-MSPH performs handover in two hops with a 13.15% success rate compared to BMAC-H, which delivers 45.38% of packets received at the MS. In the other cases (90 and 120 nodes), the MS receives data packets in a shortest end-to-end delay using CLR-MSPH compared to BMAC-H. The CLR-MSPH performs the handover in two and three hops with 20.70% and 8.04% of the total packets sent in 90 node and 120 node topologies, respectively, while BMAC-H hands over 53.33% and 37.65% of the total packets sent in 90 nodes and 120 nodes topologies, respectively. 

We justify the performance of CLR-MSPH by the efficiency of predicting and selecting the relay nodes to process the handover, even in the case where BMAC-H performs better than CLR-MSPH in terms of latency, the difference of latency is minimal as we saw in the previous test.

## 7. Conclusions

In this paper, we have studied the problem of continuous connectivity and delivering data during the data collection process by the MS. For this purpose, we have presented in this paper our proposed protocol called CLR-MSPH to deal with this problem. In order to show the performance of CLR-MSPH, we have performed simulations in different scenarios and compared the results with BMAC-H adapted to cluster-based wireless sensor networks. We see that in all studied scenarios the proposed CLR-MSPH performs better than BMAC-H in terms of packet reception rate, latency, and energy consumption. 

CLR-MSPH shows an efficient data collection even with the variation of the speed of the MS. CLR-MSPH performs in multi-hop communication (three hops) in order to route data to the MS with high packet reception rate compared to BMAC-H, which uses two hops. on the other hand, CLR-MSPH showed slightly less energy consumption than BMAC-H which proves that CLR-MSPH has an efficient energy awareness thanks to the cooperation between layers even with the multiple hop routing used by CLR-MSPH (three hops). In terms of latency (end-to-end delay), data packets are delivered with a shorter end-to-end delay using CLR-MSPH, especially with the scalability of networks.

As future work, we have other considerations to extend this work in depth and face the limitations and open issues related to the handover mechanism in cluster-based wireless sensor networks. We plan to investigate the efficiency of the proposes protocol CLR-MSPH against other protocols based on handover mechanisms such as RPL. We also plan to develop a mobility model for the MS that is adapted to the dynamic changes of network topology and CHs’ positions. Besides, studying the adjustment of the mobile sink velocity according to the cluster heads’ buffer size for a better data collection should also be considered in the mobility model of the MS. Finally, finding a suitable energy-aware clustering protocol taking into consideration the target coverage in order to organize the network in clusters and to minimize the number of the clusters, is considered one of the most important open issues that should be addressed. 

## Figures and Tables

**Table 1:** Simulation parameters.

Parameters	Values
**Simulation Time**	200 s
**Number of Nodes in topology**	30, 60, 90, 120 (Random)
**Average Number of Clusters**	5, 10, 15, 20
**Mobile Node**	Sink (MS)
**Speed**	5, 10, 15, 20, 25 m/s
**Radio Range**	50 m
**Protocol**	CLR-MSP, BMAC-H
**Packet Delivery Rate**	5 Pck/s (default)
**Node Energy**	18,720 J

**Table 2:** Simulation results in terms of packets reception rate with varying the speed of the MS.

	The Velocity of the MS in m/s.				
**Protocol**	5	10	15	20	25
**CLR-MSPH**	98.46%	97.35%	98.05%	92.17%	97.46%
**BMAC-H**	87.83%	98.21%	94.40%	88.61%	91.69%

**Table 3:** Handover packets in each protocol varying the speed of MS.

	The Velocity of the MS in m/s.				
**Protocol**	5	10	15	20	25
**CLR-MSPH 2hops**	0%	4.15%	4.95%	14.21%	10.10%
**CLR-MSPH 3hops**	0%	20.85%	19.45%	28.22%	24.59%
**BMAC-H 2hops**	68.68%	54.35%	54.11%	61.78%	39.77%

**Table 4:** The latency of packets received at the MS with varying the speed.

	The Velocity of the MS in m/s.				
**Protocol**	5	10	15	20	25
**CLR-MSPH**	77.36 s	35.33 s	30.96 s	29.49 s	21.97 s
**BMAC-H**	82.43 s	35.62 s	29.19 s	25.38 s	20.02 s

**Table 5:** Scalability and Packets Reception Rate.

	Number of Nodes in the Topology			
**Protocol**	30 nodes	60 nodes	90 nodes	120 nodes
**CLR-MSPH**	98.29%	98.05%	96.73%	94.95%
**BMAC-H**	87.25%	94.40%	94.23%	91.99%

**Table 6:** Scalability and handover packets in each protocol.

	Number of Nodes in the Topology			
**Protocol**	30 nodes	60 nodes	90 nodes	120 nodes
**CLR-MSPH 2hops**	13.15%	4.95%	7.87%	2.60%
**CLR-MSPH 3hops**	0%	19.45%	12.83%	5.44%
**BMAC-H 2hops**	45.38%	54.11%	53.33%	37.65%

**Table 7:** The latency of delivered packets in each topology.

	Number of Nodes in the Topology			
**Protocol**	30 nodes	60 nodes	90 nodes	120 nodes
**CLR-MSPH**	14.61 s	35.33 s	41.83 s	60.78 s
**BMAC-H**	18.34 s	35.61 s	42.38 s	66.11 s
